# How age-inclusive human resource practices relate to career sustainability: the role of work-family enrichment and protean career orientation

**DOI:** 10.3389/fpsyg.2025.1564719

**Published:** 2025-07-25

**Authors:** Liang Hou, Ruijing Shi, Zhongji Wang, Jun Zhang

**Affiliations:** Department of Human Resource Management and Public Administration, Beijing Institute of Petrochemical Technology, Beijing, China

**Keywords:** age-inclusive HR practices, career sustainability, work-family enrichment, protean career orientation, work-home resource model

## Abstract

Drawing on conservation of resource theory and work-home resources model, this study explores how age-inclusive human resource (HR) practices related to employees’ career sustainability through their work-family enrichment. In line with work-home resource model, we introduce a boundary condition, protean career orientation, to explain when our proposed associated may unfold. We collected data in three waves from 244 employees to test our hypotheses. Results show that age-inclusive HR practices promote employees’ career sustainability through the mediating effect of work-family enrichment. Our findings also emphasize that protean career orientation strengthens the relationship between age-inclusive HR practices and work-family enrichment. Age-inclusive HR practices were more positively linked to career sustainability via work-family enrichment when employees had a high (versus low) protean career orientation. Our research provides a better understanding of how and when age-inclusive HR practices lead to sustainable career.

## Introduction

With the global trend of workforce aging, the proportion of older employees in the labor force continues to increase (Oliveira, 2021; Kooij et al., 2020). Such as, relevant data show that by 2030, nearly half of China’s working-age population will be those over 45 years old (Peng et al., 2022). This demographic change has sparked several career-related challenges. For instance, age-related discrimination may prevent employees from staying engaged and productive (Oliveira and Cabral Cardoso, 2018; Ng and Feldman, 2012), thereby undermining their sustainable employability (Akkermans and Kubasch, 2017). From this, designing an age-inclusive work environment that fosters employees’ career sustainability, that is, a continuous sequence of career experiences over time crossing several social spaces including indicators related to well-being (e.g., health and happiness) and productivity (e.g., performance) (De Vos et al., 2020; Tordera et al., 2020), is more critical than ever.

One promising approach may be the use of age-inclusive HR practices, which emphasize having equal opportunities for employees of all age groups with regard to recruiting, training and development, promotion, and managerial support (Boehm et al., 2021). In this way, organizations can provide all employees with different resources to develop their knowledge, skills, and abilities, ultimately performing their jobs successfully and contributing to organizational goals (Kunze et al., 2015). Employees usually want to use the resources provided by the organization to improve both their work performance and non-work lives. However, most studies of age-inclusive HR practices have primarily focused on their effects on work-related outcomes (Boehm et al., 2014; Burmeister et al., 2018; Fan et al., 2023), overlooking its broader implications for career-related outcomes. Hence, it remains unknown whether age-inclusive HR practices can promote employees’ performance and personal well-being as reflecting indicators of career sustainability. These outcomes encompass both work domains (e.g., performance) and non-work domains (e.g., well-being). Furthermore, it is unclear which mechanisms underpin these relationships and produce salient effects on non-work and work domains. The current study aims to address this gap by examining the underlying processes and boundary conditions that enable age-inclusive HR practices to enhance employees’ career sustainability.

To examine *how* and *when* age-inclusive HR practices promote career sustainability, we draw on the work-home resources (WH-R) model (Ten Brummelhuis and Bakker, 2012) and conservation of resources (COR) theory (Hobfoll, 1989). According to WH-R model, contextual resources from work domains can enhance personal resources through the process of work-family enrichment, which are subsequently leveraged to improve home and work outcomes (Ten Brummelhuis and Bakker, 2012). Specifically, we propose that age-inclusive HR practices promote work-family enrichment by providing valuable resources to employees. In turn, enhanced work-family enrichment is expected to improve employees’ career sustainability, as reflected in work productivity and psychological well-being. Using a resource perspective, this study explores how age-inclusive HR practices contribute to career sustainability through the mediating effect of work-family enrichment.

Furthermore, this study investigates the boundary conditions that explain how age-inclusive HR practices and employees’ work and non-work outcomes may vary. Drawing on WH-R model, a specific type of resource—protean career orientation, which is defined as a preference for employees to self-manage their careers (Briscoe and Hall, 2006)—is considered a salient boundary condition. Most conceptions of protean career orientation implicit the whole life perspective, such as pursuing a balanced work life (Hall and Richter, 1990; Direnzo et al., 2015) and serving the whole person, family, and life purpose (Hall, 2004). Thus, we argue that protean career orientation is a meaningful key resource that may amplify the effect of age-inclusive HR practices on work-family enrichment, fostering the transfer of resources from the work domain to employees’ personal lives.

This study contributes to extant literature in several ways. First, we contribute to age-inclusive HR practices literature by extending its outcomes. Prior research has mainly focused on the impact of age-inclusive HR practices on work-related outcomes (e.g., Boehm et al., 2014; Fan et al., 2023), overlooking the role played in enabling career outcomes. By emphasizing the value and usefulness of all age groups, age-inclusive HR practices can shape employees’ perceptions of their future occupational opportunities (Oliveira, 2021). In this regard, age-inclusive HR practices may be privileged levers available to organizations that can promote sustainable careers. Our study also answers the call for attention to the role of HR practices (specifically, age-inclusive HR practices) in the development of sustainable careers (Inkson et al., 2012). Second, this study introduces a resource perspective into the age-inclusive HR practices literature. Previous studies primarily used social exchange theory (e.g., Ali and French, 2019; Sousa et al., 2019; Bieling et al., 2015; Boehm et al., 2014; Rudolph and Zacher, 2021) and socio-emotional selectivity theory (Oliveira, 2021) to explain how age-inclusive HR practices promote employees’ work-related outcomes by building a reciprocal relationship between organizations and employees. Recent studies also consider other explanation mechanisms, such as social learning theory (e.g., Fasbender and Gerpott, 2022), to explain older employees’ knowledge seeking from younger colleagues. Drawing on WH-R model and COR theory, this study investigates the impact of age-inclusive HR practices on non-work outcomes, such as work-family enrichment and career sustainability, from the perspective of resource spillover effects. Third, by identifying protean career orientation as a boundary condition that helps maximize the potential effect of age-inclusive HR practices on work-family enrichment and career sustainability, this research addresses recent calls to understand the role of individual differences in the effectiveness of age-inclusive HR practices. Although a few studies have examined the boundary conditions under which age-inclusive HR practices affect employee work outcomes (e.g., Sousa et al., 2019; Fan et al., 2023), little is known about when age-inclusive HR practices enhance or weaken employee non-work outcomes. By integrating contextual resources and key personal resources, we define protean career orientation as a boundary condition that determines the efficacy of age-inclusive HR practices in promoting work-family enrichment and sustaining employees’ careers.

### Theory and hypotheses development

#### COR theory and WH-R model

The core tenet of COR theory is that people are motivated to protect their current resources and acquire new resources (Hobfoll, 1989). Resources are loosely defined as objects, personal attributes, conditions, energies, and other things that people value. The COR theory is built on two basic assumptions (Hobfoll, 1989, 2002). The first is the *gain spiral* of resources, which posits that individuals with more resources are in a better position to invest resources and acquire more resources. The second is the *loss spiral* of resources, wherein individuals with fewer resources are more likely to experience resource losses.

The WH-R model integrates the basic assumptions of COR theory into the work-home interface (Ten Brummelhuis and Bakker, 2012). Given the important role of resources in COR theory, Ten Brummelhuis and Bakker (2012) also distinguished different types of resources in more detail. Contextual resources (e.g., age-inclusive HR practices) are external to the self and can be found in the social contexts of the individual, while personal resources (e.g., time, energies, knowledge, and skills) are proximate to the self and usually found within the individual. These distinctions assist in understanding how employees gain and utilize resources in their work environments to achieve their desired outcomes, such as career sustainability. Work-family enrichment is depicted as the process whereby contextual resources lead to the development of personal resources. To clarify the conditions that are most likely to achieve work-home enrichment, the WH-R model proposes key resources and macro resources. The former refers to stable personal characters that facilitate the selection, alteration, and implementation of other resources (e.g., conscientiousness; Halbesleben et al., 2009; Penney et al., 2011). The latter refers to characteristics of the larger economic, social, and cultural system in which a person is embedded (Halbesleben et al., 2014). Contextual resources do not affect all individuals equally, and people with key resources are more likely to effectively leverage their contextual resources to gain personal resources (Ten Brummelhuis and Bakker, 2012).

Drawing on the WH-R model, this study explores how age-inclusive HR practices, as a type of contextual resource in the workplace, can enhance employees’ career sustainability through the process of work-home enrichment. Furthermore, this study examines whether protean career orientation, conceptualized as a key resource, serves as a boundary condition that influences this relationship, thereby testing the applicability of the WH-R model.

#### Age-inclusive HR practices and work-family enrichment

Work-family enrichment is defined as the degree to which role experiences and resources from the work domain enhance the quality of life in the family domain (Greenhaus and Powell, 2006). From the perspective of the WH-R model, work-family enrichment can be understood as a process in which contextual resources from the work domain are translated into personal resources (Ten Brummelhuis and Bakker, 2012). Specifically, work-family enrichment occurs through two pathways: the instrumental path and the affective path (Greenhaus and Powell, 2006). The instrumental path involves the acquisition of direct resources at work, such as skills, experiences, and social capital, which directly enhance family role performance. The affective path, on the other hand, refers to resources obtained at work that foster one’s positive emotions, which in turn improve outcomes in the family domain (Lapierre et al., 2018).

Age-inclusive HR practices refer to effective management practices designed to accommodate the growing workforce aging (Boehm et al., 2014). In line with the WH-R model, these practices are posited to influence employees’ work-family enrichment through two pathways. First, through the instrumental pathway, age-inclusive HR practices provide employees with equitable access to training, promotion, and career development opportunities regardless of their age (Hennekam and Herrbach, 2015; Boehm et al., 2021). Resources acquired through these practices, such as enhanced skills or increased compensation, can directly improve employees’ functioning in family role. For instance, skills developed through training may help resolve both work-related challenges and similar family issues (Greenhaus and Powell, 2006), while higher compensation can better support household expenses, thereby improving the quality of life at home (Heinrich, 2014). Second, through affective pathway, age-inclusive HR practices address age discrimination issues by ensuring fair treatment for employees of all ages (Fan et al., 2023; Li et al., 2021; Boehm et al., 2014). This fosters feelings of being valued and supported, thereby enhancing employees’ affective commitment to their organization (Fan et al., 2023; Sousa et al., 2021). These positive emotions can spill over into family domain, improving family-related outcomes (Chan et al., 2022). Supporting these arguments, previous studies have demonstrated that contextual resources from the work domain can enhance work-family enrichment through both instrumental and affective pathways (e.g., Hammond et al., 2015; Liu et al., 2024). Thus, we propose the following hypothesis:

*Hypothesis 1*: Age-inclusive HR practices are positively related to work-family enrichment.

### Work-family enrichment and career sustainability

Career sustainability is defined as “sequences of career experiences reflected through a variety of patterns of continuity over time, thereby crossing several social spaces, characterized by individual agency, herewith providing meaning to the individual” (Van der Heijden and De Vos, 2015). Health, happiness, and productivity are the three elements that constitute sustainable careers (De Vos et al., 2020). Building on this framework, Tordera et al. (2020) refined two dimensions — *well-being* (health and happiness) and *productivity* (performance)—to be the reliable indicators for career sustainability. Well-being and work performance have also been regarded as important non-work and work outcomes for employees (Warr and Nielsen, 2018; Kong et al., 2023).

A sustainable career across the lifespan requires individuals to invest personal resources and balance interests across multiple social domains, such as work and family (De Vos et al., 2020). According to COR theory and WH-R model, the value of resources is that it helps one achieve his or her goals, thereby enhancing one’s well-being and performance (Wright and Hobfoll, 2004; Panaccio and Vandenberghe, 2009). In terms of well-being, work-family enrichment facilitates the transfer of resources and skills between work and family domains, extending the positive experience at work to the family and life domains (Liu et al., 2018; Hunter et al., 2010; Jing et al., 2021). These aspects will enhance life satisfaction and well-being (Henry and Desmette, 2018). Moreover, work-family enrichment can increase one’s positive mood in the form of enthusiasm and energy, enabling the individual a sense of self-fulfillment (Carlson et al., 2011). Overall, work-family enrichment meets employees’ psychological needs, thereby improving their satisfaction and well-being (Chan et al., 2020; Baumann and Wilson, 2024).

In terms of productivity, work-family enrichment can place employees in a resource-abundant state, which enhances their capability and willingness to engage in productive work behaviors. When employees accumulate resources in the family role due to work-family enrichment, they are more likely to generate a sense of reciprocity toward the organization and commit to reinvesting resources into the work role, resulting in improved productivity (Ten Brummelhuis and Bakker, 2012). Second, skills, knowledge, behaviors, and perspective obtained from one’s experiences at work and family life are resources that generates benefits across life domains (Carlson et al., 2006). For instance, a sense of focus or necessity to sustain work in order to support a family can also be described as a resource that enables employees to be better workers. Therefore, in line with WH-R model, we predict that these resources will demonstrate gains in the form of work engagement and work performance (Timms et al., 2015; Carlson et al., 2011). Overall, work-family enrichment enhances individuals’ competencies and motivational resources for work, promoting sustainable careers. Taken together, we propose the following hypothesis:

*Hypothesis 2:* Work-family enrichment is positively related to employees’ career sustainability (well-being and productivity).

In summary, we argue that age-inclusive HR practices positively related to career sustainability through the mediating effect of work-family enrichment. Based on COR theory and the WH-R model, gaining contextual resources lead to the accumulation of personal resources (i.e., work-family enrichment), which in turn re-invest their resources and efforts in the work and life domains. The argument is further supported by prior studies, which indicate that work-family enrichment is associated with increased well-being and productivity (McNall et al., 2010; Odle-Dusseau et al., 2012). We thus hypothesize an indirect effect of age-inclusive HR practices on career sustainability through work-family enrichment.

*Hypothesis 3:* Work-family enrichment mediates the relationship between age-inclusive HR practices and employees’ career sustainability (well-being and productivity).

### The moderating role of protean career orientation

Protean career orientation (PCO) refers to an individual’s tendency to achieve subjective success through proactive career management, including two dimensions: self-direction and value-driven orientation (Hall et al., 2018). Self-direction is manifested in individuals’ continuous learning and strengthening of their capabilities to achieve their career goals (Cortellazzo et al., 2020), accompanied by a strong desire for autonomy and self-actualization (Li et al., 2019). Value-driven orientation refers to the pursuit of career goals that align with one’s intrinsic values (Li et al., 2019). Clear values guide individuals to manage their careers with a more holistic and long-term perspective (Briscoe and Hall, 2006; Cortellazzo et al., 2020).

The self-directed and value-driven orientation inherent in PCO equips individuals with stronger capability and motivation to control and select resources, making it a key resource (Direnzo et al., 2015). Based on the WH-R model, we propose that PCO, as a key resource, enables individuals to better utilize contextual resources in the work domain (i.e., age-inclusive HR practices) to achieve work-family enrichment. On one hand, PCO are self-directed and can proactively scan and reshape external environment around them to seek resources that meet its expectations (Hall et al., 2018; Cortellazzo et al., 2020). Therefore, individuals with high PCO are better at identifying and leveraging both the instrumental and emotional resources that age-inclusive HR practices provide, thereby enhancing work-family enrichment. On the other hand, individuals with high PCO adopt a holistic perspective on career development (Direnzo et al., 2015; Direnzo and Greenhaus, 2011). They view career development not as a process limited to self-development within the work domain, but as a comprehensive form of self-development that spans all life domains (Direnzo et al., 2015; Hall and Chandler, 2005). Thus, individuals with high levels of PCO are more motivated to transfer the resources identified in the work domain to the life domain, thereby promoting enrichment across both work and family domains.

However, individuals with low levels of PCO may lack the proactivity and awareness to pursue opportunities, making it difficult for them to effectively utilize potential resources in the organizational environment. Additionally, individuals with low PCO may lack value-driven guidance, making it challenging for them to recognize the importance and prioritize matters related to career development. As a result, they may become dependent on and controlled by the external environment, without a sufficient perspective to guide their own career development, thereby hindering their ability to achieve work-family enrichment (Briscoe and Hall, 2006). Based on this, we propose the following hypothesis:

*Hypothesis 4:* Protean career orientation moderates the positive relationship between age-inclusive HR practices and work-family enrichment, such that this relationship is stronger when protean career orientation is higher (versus lower).

**Figure 1:**
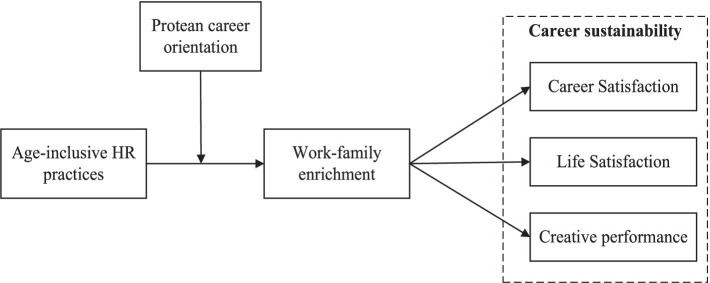
Hypothesized research model.

Based on the COR theory and the WH-R model, we further propose that PCO moderates the indirect effect of work-family enrichment between age-inclusive HR practices and career sustainability. Individuals with high level of PCO are guided to proactively identify resources and support provided by age-inclusive HR practices and to invest these resources across work and family domains with a long-term perspective, thereby achieving comprehensive enrichment and maintaining career sustainability. Conversely, individuals with low level of PCO often lack proactivity and may struggle to uncover potential applicable resources within organizational support. Due to a lack of a holistic and long-term developmental perspective, they find it challenging to convert available resources into enrichment across multiple domains such as work and family, which hampers their ability to plan and manage their careers systematically (Briscoe and Hall, 2006). Based on the above arguments, we offer the following model (see Figure 1).

*Hypothesis 5:* The indirect effect of work-family enrichment between age-inclusive HR practices and career sustainability is stronger when protean career orientation is higher (versus lower).

## Methods

### Sample and procedures

We collected our data from knowledge employees working at various organizations in China. One of the main reasons is that the core competitiveness of knowledge workers depends on continuous knowledge updating and experience accumulation. Compared with manual laborers, their career life cycle is longer, and they face a more significant risk of knowledge aging. Thus, this study mainly uses a sample of knowledge workers to test the relationship between age-inclusive HR practices and career sustainability. We recruited participants in the Credamo platform^1^, which has been commonly used in organizational studies (e.g., Cheng et al., 2024; Huang et al., 2023). During the data collection process, we informed all participants that this survey would be used for research purposes only and that their responses would be kept strictly confidential. In order to increase the response rate, we paid each participant the bottom-line compensation set by the platform.

In order to better identify the causal relationships among variables and avoid the effects of common method biases, we conducted a three-wave data collection process, with a two-week interval. At the first time point (Time1), participants were asked to fill out a relatively comprehensive questionnaire measuring variables including demographic variables (i.e., gender, age, education, and tenure), age-inclusive HR practices, protean career orientation, and initial level of career sustainability (well-being and productivity). Initially, we distributed a total of 400 questionnaires. We used a screening question to excluding those who did not answer truthfully, finally receiving 339 valid employee questionnaires, a response rate of 84.8%. Two weeks later, at Time 2, we asked these 339 employees to rate their work-family enrichment. A total of 296 employees completed their questionnaires, generating a response rate of 87.3%. At Time 3, we invited 296 employees to report the level of career sustainability (well-being and productivity) and obtained 244 responses, generating a response rate of 82.4%. Finally, we matched 244 responses with the three-wave data using the platform’s user IDs.

Among these employees, 31.1% were male and 68.9% were female; the average age of participants was 33.69 years (SD = 7.22, with a missing value); the average organizational tenure was 6.21 years (SD = 4.72). In terms of education, most had undergraduate degrees (59%), while 23.1% had postgraduate degrees and above, 15.6% had college degrees, and 4.1% had high school qualifications.

### Measures

In our study, we measured all variables using well-established scales that were originally developed in English. We translated these scales into Chinese using the back-translation procedure (Brislin, 1970). Unless otherwise stated, all items were measured by a 5-point Likert scale (from 1 = *strongly disagree* to 5 = *strongly agree*).

### Age-inclusive HR practices

We measured age-inclusive HR practices using a five-item scale developed by Boehm et al. (2014). The five items of this scale measured the degree to which employees perceived age-inclusive HR practices in their organization. An example item was “our company offers equal access to training and further education for all age groups.” The Cronbach’s alpha in this study was 0.87.

### Protean career orientation

A seven-item scale developed by Baruch (2014) was used to assess employees’ protean career orientation. One example item was “For me, career success is how I am doing against my goals and values.” The Cronbach’s alpha for this scale was 0.84.

### Work-family enrichment

Employees rated their work-family enrichment at Time 2 using a four-item scale established by Wayne et al. (2004). One example was “The things you do at work help you deal with personal and practical issues at home.” The Cronbach’s alpha was 0.90.

### Career sustainability

According to De Vos et al.’s (2020) conceptual model, we used life satisfaction, career satisfaction, and creative performance as key indicators of career sustainability. Well-being is typically measured by individuals’ perceptions of life satisfaction and career satisfaction (e.g., Kong et al., 2023; Tordera et al., 2020). Specifically, we measured career satisfaction with a five-item scale developed by Greenhaus et al. (1990). One of example items was “I am satisfied with the success I have achieved in my career” (*α* = 0.92). We used a five-item scale developed by Diener et al. (1985) to measure life satisfaction. Participants were asked to indicate how satisfied they were with their lives. Typical an item was “In most ways, my life is close to my ideal” (α = 0.93). In terms of productivity, Baer and Oldham’s (2006) 4-item scale were used to measure employee creative performance. An example item was “I often come up with creative solutions to problems at work” (α = 0.88).

### Control variables

We controlled for gender, age, education and organizational tenure owing to their established relationships with career satisfaction, life satisfaction and creative performance (e.g., Ng and Feldman, 2013; Jiang and Hu, 2016; Kong et al., 2022; Solomon et al., 2022). Furthermore, in line with previous research (e.g., Ren et al., 2024), to verify that age-inclusive HR practices have incremental effects on career sustainability above prior levels, we also controlled for the level of career sustainability at Time 1. We assessed career satisfaction (α = 0.92), life satisfaction (α = 0.90), and creative performance (α = 0.88) using the same scales as Time 3. We note that removing these controls does not affect the statistical significance of our findings. In order to rigorously test the hypothesis, we report the results of including these control variables.

### Analytical strategy

To test our hypotheses, we conducted path analysis using Mplus 8.3 (Muthén and Muthén, 2019). To examine the mediating effects and moderated mediation effects, we used the Monte Carlo simulations with 20,000 replications to calculate the 95% confidence intervals (Selig and Preacher, 2008). When confidence intervals do not contain zero, the indirect association was significant. To examine the moderating effects, we grand-mean centered all independent variables (Cohen et al., 2003). Then, we plotted simple slopes at one standard deviation below and above the mean of the moderator to interpret our results (Aiken and West, 1991).

## Results

Table 1 presents the results of descriptive statistics and correlations. As shown, A high level of education made it easier for employees to have career satisfaction (*r* = 0.19, *p* < 0.01), life satisfaction (*r* = 0.21, *p* < 0.01) and creative performance (*r* = 0.30, *p* < 0.001). Organizational tenure was positively related to work-family enrichment (*r* = 0.13, *p* < 0.05). Age-inclusive HR practices had a positive and significant relationship with work-family enrichment (*r* = 0.29, *p* < 0.001). Similarly, work-family enrichment was positively related to employees’ career satisfaction (*r* = 0.36, *p* < 0.001), life satisfaction (*r* = 0.36, *p* < 0.001), and creative performance (*r* = 0.26, *p* < 0.001). These results provided preliminary support for hypothesis testing.

**Table 1:** Mean, standard deviations, and zero-order correlations.

Variables	*M*	SD	1	2	3	4	5	6	7	8	9	10	11	12	13
1 Gender	0.69	0.46													
2 Age	33.69	7.22	−0.03												
3 Education	2.98	0.73	0.07	−0.26***											
4 Organizational tenure	6.21	4.72	−0.15*	0.50***	−0.14*										
5 Career satisfaction (T1)	3.88	0.91	0.01	0.12	0.15*	0.12	**(0.92)**								
6 Life satisfaction (T1)	3.67	0.93	0.07	0.13*	0.11	0.12	0.78***	**(0.90)**							
7 Creative performance (T1)	3.93	0.78	−0.02	0.14*	0.14*	0.07	0.70***	0.57***	**(0.88)**						
8 Protean career orientation (T1)	4.11	0.61	0.001	−0.02	0.20**	−0.02	0.59***	0.52***	0.67***	**(0.84)**					
9 Age-inclusive HRM (T1)	3.94	0.84	0.01	−0.06	0.20**	−0.01	0.69***	0.61***	0.63***	0.71***	**(0.87)**				
10 Work-family enrichment (T2)	3.77	0.92	−0.02	0.02	−0.02	0.13*	0.35***	0.41***	0.30***	0.15*	0.29***	**(0.90)**			
11 Career satisfaction (T3)	3.92	0.86	−0.03	0.05	0.19**	0.09	0.55***	0.53***	0.41***	0.30***	0.40***	0.36***	**(0.92)**		
12 Life satisfaction (T3)	3.59	0.97	0.08	0.08	0.21**	0.11	0.50***	0.62***	0.33***	0.23***	0.36***	0.36***	0.77***	**(0.93)**	
13 Creative performance (T3)	3.99	0.79	−0.002	0.09	0.30***	0.12	0.46***	0.40***	0.47***	0.28***	0.39***	0.26***	0.74***	0.73***	**(0.88)**

*N* = 244; Alpha reliabilities appear in the parentheses along diagonal. SD = standard deviation; Gender is code as “0 = man, 1 = woman”; Education is code as “1 = high school and below, 2 = junior college diploma, 3 = bachelor, 4 = master and above.” *p < 0.05, **p < 0.01, ***p < 0.001.

Alpha reliabilities appear in parentheses along the diagonal and are in bold.

### Test of measurement model

To test discriminant validity, we conducted a confirmatory factor analysis (CFA) on six focal variables (i.e., age-inclusive HR practices, protean career orientation, work-family enrichment, career satisfaction, life satisfaction, and creative performance). As shown in the Table 2, results suggested that the hypothesized six-factor model fits the data well (χ^2^ = 829.01, df = 390, CFI = 0.92, TLI = 0.91, RMSEA = 0.07, SRMR = 0.06) and better than any of the five-factor models.

**Table 2:** Model fit results for confirmatory factor analyses.

Models	χ^2^(df)	(df)	*χ^2^/*df	CFI	TLI	RMSEA	SRMR
Hypothesized six-factor model	829.01	390		0.92	0.91	0.07	0.06
Five-factor model (combing AHRP and protean career orientation)	917.12	395	88.11/5***	0.90	0.89	0.07	0.07
Five-factor model (combing AHRP and work-family enrichment)	1440.25	395	611.24/5***	0.80	0.78	0.10	0.10
Five-factor model (combing protean career orientation and work-family enrichment)	1486.69	395	657.68/5***	0.79	0.77	0.11	0.11
Five-factor model (combing career satisfaction and life satisfaction)	1002.50	395	173.49/5***	0.88	0.87	0.08	0.07
Five-factor model (combing work-family enrichment and creative performance)	1534.61	395	705.60/5***	0.78	0.76	0.11	0.16

**△ =** change relative to the measurement model; CFI = comparative fit index; TLI = Tucker-Lewis index; RMSEA = root mean squared error of approximation; SRMR = standardized root mean-square residual. ***p < 0.001.

### Testing the hypotheses

We performed path analysis to test our hypotheses using Mplus 8.3 software and the results were summarized as Table 3. Age-inclusive HR practices was positively associated with work-family enrichment (*B* = 0.45, *SE* = 0.10, *p* < 0.001). Thus, Hypothesis 1 was supported. In addition, work-family enrichment had a significantly positive effect on employees’ career satisfaction (*B* = 0.21, *SE* = 0.05, *p* < 0.001), life satisfaction (*B* = 0.18, *SE* = 0.06, *p* < 0.01) and creative performance (*B* = 0.11, *SE* = 0.05, *p* < 0.05). These results supported hypotheses 2 that work-family enrichment had positive relationships with employee career sustainability.

**Table 3:** Unstandardized coefficients of path model.

Variables	Work-family enrichment		Career satisfaction		Life satisfaction		Creative performance	
	Estimate	*SE*	Estimate	*SE*	Estimate	*SE*	Estimate	*SE*
Gender	0.01	0.12	−0.08	0.10	0.10	0.11	0.01	0.09
Age	−0.01	0.01	0.01	0.01	0.01	0.01	0.004	0.01
Education	−0.06	0.08	0.17**	0.07	0.24***	0.07	0.28***	0.06
Organizational tenure	0.03	0.01	0.002	0.01	0.01	0.01	0.02	0.01
Career satisfaction (T1)			0.34***	0.05				
Life satisfaction (T1)					0.46***	0.05		
Creative performance (T1)							0.30***	0.05
AHRP (T1)	0.45***	0.10	0.06	0.07	0.02	0.07	0.11	0.06
PCO (T1)	−0.05	0.14						
AHRM*PCO	0.19**	0.07						
Work-family enrichment (T2)			0.21***	0.05	0.18**	0.06	0.11*	0.05
*R^2^*	0.14**	0.04	0.31***	0.05	0.36***	0.05	0.30***	0.05

*N* = 244; AHRP = Age-inclusive human resource practices; PCO = protean career orientation. *p < 0.05, **p < 0.01, ***p < 0.001.

In support of Hypothesis 3, we used the Monte Carlo simulations with 20,000 replications to calculate the 95% confidence intervals. Results showed that the indirect effect of age-inclusive HR practices on employee career satisfaction (indirect effect = 0.09, *SE* = 0.03, 95% CI = [0.04, 0.17]), life satisfaction (indirect effect = 0.08, *SE* = 0.03, 95% CI = [0.03, 0.15]), and creative performance (indirect effect = 0.05, *SE* = 0.02, 95% CI = [0.01, 0.11]) through work-family enrichment were positive and significant. The result suggested that work-family enrichment mediated the relationship between Age-inclusive HR practices and career sustainability, supporting hypothesis 3.

**Figure 2:**
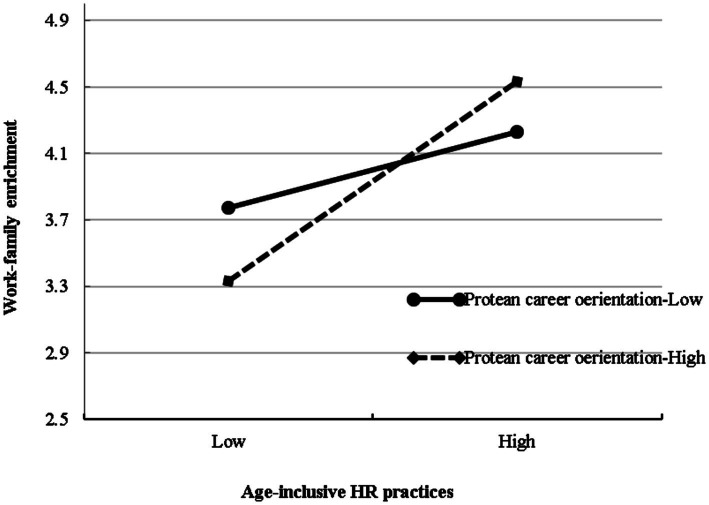
Interaction between age-inclusive HR practices and protean career orientation on work-family enrichment.

Hypothesis 4 proposes that protean career orientation moderates the relationship between age-inclusive HR practices and work-family enrichment. Results showed that the interaction term between age-inclusive HR practices and protean career orientation had a significant effect on work-family enrichment (*B* = 0.19, *SE* = 0.07, *p* < 0.01), providing initial support for the moderating of protean career orientation. We further plotted the interaction at conditional values of protean career orientation (Figure 2). For the relationship between age-inclusive HR practices and work-family enrichment, the slope was stronger when employees’ protean career orientation was higher (simple slope = 0.57, *p* < 0.001) rather than lower (simple slope = 0.34, *p* < 0.01). The difference in simple slope between the two conditions was significant (*p* < 0.01). Therefore, Hypothesis 4 was supported.

We also examined the conditional indirect effects of age-inclusive HR practices on career sustainability through work-family enrichment at varying levels of protean career orientation (one SD above the mean and one SD below the mean). As shown in Table 4, the results indicated that the conditional indirect effects of age-inclusive HR practices via work-family enrichment on employees’ career satisfaction were significant at both high (indirect effect = 0.12, *SE* = 0.04, 95% CI = [0.06, 0.21]) and low (indirect effect = 0.07, *SE* = 0.03, 95% CI = [0.03, 0.14]) levels of protean career orientation. The conditional indirect effect of age-inclusive HR practices via work-family enrichment on employees’ life satisfaction was significantly positive at both high (indirect effect = 0.10, *SE* = 0.04, 95% CI = [0.04, 0.19]) and low (indirect effect = 0.06, *SE* = 0.03, 95% CI = [0.02, 0.12]) levels of protean career orientation. The conditional indirect effect of age-inclusive HR practices via work-family enrichment on employees’ creative performance was significantly positive at both high (indirect effect = 0.06, *SE* = 0.03, 95% CI = [0.004, 0.14]) and low (indirect effect = 0.04, *SE* = 0.02, 95% CI = [0.003, 0.10]) levels of protean career orientation. The differences in the indirect effects between the two conditions were 0.05 (career satisfaction), 0.04 (life satisfaction), 0.03 (creative performance) with a 95% bias-corrected CI of (0.01, 0.10), (0.01, 0.09), and (0.004, 0.07), respectively. Thus, Hypothesis 5 was supported.

**Table 4:** Moderated mediation results.

Outcomes variables	Levels of protean career orientation	Indirect effect	SE	95% bias-corrected CI	Difference of effects and 95% bias-corrected CI
Career satisfaction	Low (–1SD)	0.07	0.03	[0.03, 0.14]	0.05 [0.01, 0.10]
	High (+1SD)	0.12	0.04	[0.06, 0.21]	
Life satisfaction	Low (–1SD)	0.06	0.03	[0.02, 0.12]	0.04 [0.01, 0.09]
	High (+1SD)	0.10	0.04	[0.04, 0.19]	
Creative performance	Low (–1SD)	0.04	0.02	[0.003, 0.10]	0.03 [0.004, 0.07]
	High (+1SD)	0.06	0.03	[0.004, 0.14]	

*N* = 244; SE = standard error; CI = confidence intervals. *p < 0.05, **p < 0.01, ***p < 0.001.

**Figure 3:**
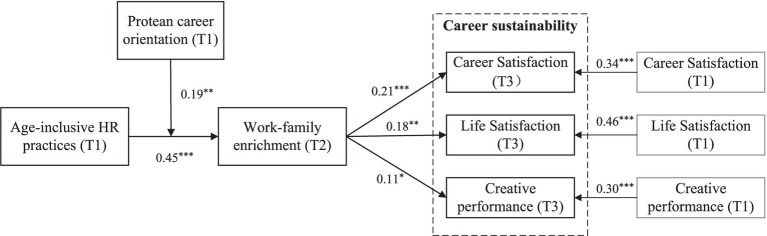
Unstandardized estimates of path coefficients. Effects of the control variables and direct effects between age-inclusive HR practices and career sustainability are not included for the purpose of clarity. *p < 0.05, **p < 0.01, ***p < 0.001.

Figure 3 summarizes the results of hypotheses paths, including direct and indirect effects.

## Discussion

This study provides empirical evidence concerning the mechanism through which age-inclusive HR practices relate to employees’ career sustainability. Our results show that age-inclusive HR practices are not only directly associated with career sustainability, but also relate to career sustainability via work-family enrichment. This finding extends an assumption of social exchange theory, which suggest that age-inclusive HR practices make employees feel support from organizations, and in exchange, employees of all ages will tend to reciprocate the organizational support through beneficial work attitudes and behaviors (Ali and French, 2019; Sousa et al., 2019; Bieling et al., 2015; Rudolph and Zacher, 2021). However, recent studies have suggested that reciprocity is not the only mechanism through which to understand the effects of age-inclusive HR practices on employees’ outcomes (e.g., Sousa et al., 2019; Oliveira, 2021; Fasbender and Gerpott, 2022). The current study introduces a resource respective into age-inclusive HR practices literature, which reveals the mechanism of age-inclusive HR practices on career sustainability (well-being and productivity). Results show that work-family enrichment plays an important role in linking age-inclusive HR practices to better career satisfaction, life satisfaction, and creative performance.

Moreover, our study shows that protean career orientation moderates the indirect effect of work-family enrichment between age-inclusive HR practices and career sustainability. The results indicates that for employees with a high level of protean career orientation, age-inclusive HR practices are more positively related to career sustainability via work-family enrichment. Prior research has emphasized that protean career orientation can directly link to a wide range of employees’ career outcomes (e.g., Baruch et al., 2020; Cortellazzo et al., 2020; Herrmann et al., 2015) and work-life balance (e.g., Direnzo et al., 2015). Considering with the contextual resources and personal key resources interaction perspective, we identified protean career orientation as a moderator that strengthens the positive effects of age-inclusive HR practices on work-family enrichment and career sustainability, which enriches the boundary condition of personal attributes in WH-R model.

### Theoretical implications

This study has several theoretical contributions to literature about age-related HR practices and sustainable career. First, our study extends the age-related HRM literature by demonstrating a positive association between age-inclusive HR practices and career sustainability. Although scholars have showed the significant role played by age-inclusive HR practices in predicting organizational and individual outcomes (Boehm et al., 2014; Fan et al., 2023), the relationship between age-inclusive HR practices and career outcomes remains unclear. By identifying career sustainability as a critical outcome of age-inclusive HR practices, this study responds to the call to focus on the role of HR practices (specifically, age-inclusive HR practices) in the development of sustainable careers (Inkson et al., 2012).

Second, we extend previous research to understand the impact mechanism of age-inclusive HR practices on desirable outcomes by introducing a resource perspective. Previous studies have mainly used social exchange theory to explain the impact of age-inclusive HR practices (Ali and French, 2019). Nevertheless, social exchange relationships usually explain the reciprocity between employees and organization, and it remains unknown how age-inclusive HR practices benefit employees outside the organization. Combing the WH-R model and COR theory, contextual resources from the work domain (i.e., age-inclusive HR practices) are effective in enabling individuals to achieve home-related and career-related goals (Halbesleben, 2006). In line with this stream of research, we found that work-family enrichment helps employees to gain personal resources, thus benefiting their well-being and productivity. Therefore, beyond the understanding of the relationship between age-inclusive HR practices and work outcomes, it is also important to consider the spillover effects of resources, which explain how work-family enrichment associated with age-inclusive HR practices affects career sustainability.

Third, this study contributes to the HRM literature by showing how age-inclusive HR practices interacts with employees’ protean career orientation to influence work-family enrichment. Although prior studies demonstrated that protean career orientation directly links to work-life balance (e.g., Direnzo et al., 2015), its boundary role in the relationship between HR practices and their work-family outcomes is unexplored. From a whole life perspective, protean career orientation emphasizes more than an orientation toward work but rather extends to other domain in an individual’s life, such as family domain (Direnzo et al., 2015). Therefore, it is pivotal to consider career orientation when exploring the effects of age-inclusive HR practices on work-family enrichment. Based on WH-R model, resources are essential to effective functioning in different life domains (Ten Brummelhuis and Bakker, 2012). Employees with high protean career orientation can facilitates one’s ability to transfer resources across different life spaces. Considering the interaction perspective of contextual resources and key resources, we identified protean career orientation as a moderator that strengthens the positive effect of age-inclusive HR practices on work-family enrichment, which enriches the boundary condition of personal attributes in WH-R model.

### Practical implications

Our findings have some practical implications. We found that age-inclusive HR practices are significantly related to work-family enrichment and, subsequently, to sustainable careers. Hence, the value of age-inclusive HR practices should be recognized by organizations and managers. Organizations, particularly those with diverse age groups, could provide equal opportunities in recruitment, promotion, and career development for all employees, regardless of their age. These practices are beneficial to the development of both employees and their organizations.

Second, our findings support that work-family enrichment plays a gain process in personal resources. For organizations, recognizing that employees are individuals with multiple identities may be more beneficial in achieving both individual and organizational long-term goals. Specifically, work-family enrichment, as a prime example of positive interaction between work and family life, holds significant value and deserves attention. Moreover, organizations and managers should also implement timely interventions to manage the process of realizing work-family enrichment, such as providing resources related to family support. Notably, research indicates that training supervisors to be more supportive of employees’ nonwork life is an effective approach for improving employee work, family, and health outcomes (Russo et al., 2018; Crain and Stevens, 2018).

Third, the relationship between age-inclusive HR practices and work-family enrichment was stronger for employees with high protean career orientation. This suggests that managers should be concerned about their subordinates’ career orientation when conducting age-inclusive HR practices. Supervisors should notice the value of protean career orientation in such an age-diverse work environment and support employees to self-manage their careers. Leaders also need to pay attention to those employees who have been embedded in high protean career orientation, which helps them to select adaptable employees as role models and effective assets within companies. These employees may play positive roles in promoting work-family enrichment and maintaining career sustainability in the age diverse organizational context.

## Limitations and future directions

Despite its strengths, this study has several limitations. First, although the multi-wave design of this study can reduce common method bias, it cannot make accurate causal inferences. That is, more caution is needed in interpreting the relationship between age-inclusive HR practices and career sustainability. We encourage future research to adopt experimental designs or longitudinal studies to address this limitation. In addition, future research can also use longitudinal research designs to examine the impact of career sustainability on employees’ subsequent work outcomes (such as psychological contract, work engagement, and organizational belonging). Given the reciprocal nature of resources in the work and family domains as described by the WH-R model, future studies could simultaneously examine how contextual resources in the work domain affect career sustainability and, conversely, how career sustainability impacts work-domain outcomes. This approach would provide a more comprehensive understanding of the interplay between these factors. Second, this study focuses on the role played by protean career orientation in the relationship between age-inclusive HR practices and work-family enrichment. There might be other potential contextual factors, for example, inclusive leadership (Carmeli et al., 2010; Javed et al., 2019) and macro culture, that will also moderate the effects of age-inclusive HR practices on employees’ reactions. Third, the survey sample of this study is mainly for knowledge workers, and manual workers are not considered. There may be significant differences in career development and future expectations between knowledge workers and manual workers. Future research can further expand and test the conclusions of this study in a larger sample.

## Data Availability

The raw data supporting the conclusions of this article will be made available by the authors, without undue reservation.

## Footnotes

^1^ https://www.credamo.com/
